# Protocol for the Delirium and Cognitive Impact in Dementia (DECIDE) study: A nested prospective longitudinal cohort study

**DOI:** 10.1186/s12877-017-0479-3

**Published:** 2017-04-28

**Authors:** Sarah J. Richardson, Daniel H.J. Davis, Blossom Stephan, Louise Robinson, Carol Brayne, Linda Barnes, Stuart Parker, Louise M. Allan

**Affiliations:** 1 Institute of Neuroscience, 3rd floor Biomedical Research Building, Newcastle University Campus for Ageing and Vitality, Newcastle upon Tyne, NE4 5PL UK; 2 MRC Unit for Lifelong Health and Ageing, University College London, 33 Bedford Place, London, WC1B 5JU UK; 3 Institute for Ageing, 2nd floor Biomedical Research Building, Newcastle University Campus for Ageing and Vitality, Newcastle upon Tyne, NE4 5PL UK; 4 Institute of Public Health, Forvie Site, School of Clinical Medicine, University of Cambridge, Cambridge Biomedical Campus, Cambridge, CB2 0SR UK

**Keywords:** Delirium, Dementia, Cognitive outcomes, Cohort, CFAS II

## Abstract

**Background:**

Delirium is common, affecting at least 20% of older hospital inpatients. It is widely accepted that delirium is associated with dementia but the degree of causation within this relationship is unclear. Previous studies have been limited by incomplete ascertainment of baseline cognition or a lack of prospective delirium assessments. There is an urgent need for an improved understanding of the relationship between delirium and dementia given that delirium prevention may plausibly impact upon dementia prevention. A well-designed, observational study could also answer fundamental questions of major importance to patients and their families regarding outcomes after delirium.

The Delirium and Cognitive Impact in Dementia (DECIDE) study aims to explore the association between delirium and cognitive function over time in older participants. In an existing population based cohort aged 65 years and older, the effect on cognition of an episode of delirium will be measured, independent of baseline cognition and illness severity. The predictive value of clinical parameters including delirium severity, baseline cognition and delirium subtype on cognitive outcomes following an episode of delirium will also be explored.

**Methods:**

Over a 12 month period, surviving participants from the Cognitive Function and Ageing Study II-Newcastle will be screened for delirium on admission to hospital. At the point of presentation, baseline characteristics along with a number of disease relevant clinical parameters will be recorded. The progression/resolution of delirium will be monitored. In those with and without delirium, cognitive decline and dementia will be assessed at one year follow-up. We will evaluate the effect of delirium on cognitive function over time along with the predictive value of clinical parameters.

**Discussion:**

This study will be the first to prospectively elucidate the size of the effect of delirium upon cognitive decline and incident dementia. The results will be used to inform future dementia prevention trials that focus on delirium intervention.

## Background

Delirium is a severe neuropsychiatric syndrome of brain dysfunction precipitated by acute illness. It is characterised by acute and fluctuating inattention and other cognitive and perceptual deficits.

Delirium is common, affecting at least 20% of older hospital inpatients [1]. Delirium is particularly common in older people and those with cognitive impairment. The occurrence rate of delirium in general medical and geriatric medicine wards was calculated at 29–64% [2]. However, delirium can occur in people of any age if the physiological insult is great enough and affects up to 80% of those in intensive care [3].

Delirium is highly unpleasant and frightening for patients and their families, causing considerable short and long-term distress [4]. People who have delirium during their hospitalisation have increased lengths of stay [5], more hospital-acquired complications, such as falls and pressure sores, are more likely to need to be admitted to long-term care following discharge from hospital and are more likely to die [6]. These complications lead to additional healthcare costs, estimated at an extra £13,000 per admission [7].

Emerging literature indicates that delirium is a strong predictor of new-onset dementia as well as acceleration of existing cognitive decline [8–11]. This is consistent across different settings: after hospitalisation [6]; in those with dementia [8, 12]; in post-operative patients [13, 14]; after critical care [11]; and in community populations [10, 15]. The only population-based study showed an 8 fold increased risk of cognitive decline following an episode of delirium [10]. Acute hospitalisation itself has been shown to adversely affect trajectories of cognitive decline, even when delirium has not been specifically ascertained [16–18]. This implies that delirium and/or its acute causes can contribute to the overall burden of dementia.

Delirium was previously thought to be a benign and transient condition and, consequently, is under-researched, well out of proportion to its prevalence and impact. The few studies that do exist have several key limitations.

In existing delirium research, good quality, large epidemiological studies in unselected populations are lacking which introduces selection bias and limits the generalisability of results [19]. Dementia is a major risk factor for delirium but many delirium studies list dementia as an exclusion criterion, potentially resulting in an underestimation of the true incidence of delirium.

It is likely that previous studies may have been confounded by incomplete ascertainment of cognitive status at baseline [10], particularly given that around half of dementia is undiagnosed [20]. Only one study of cognitive trajectories in delirium has included baseline cognitive assessments [10]. The major limitation of this work was that delirium could not be prospectively defined. There is a clear case for using prospective delirium assessments particularly in the context of a cohort study as this would allow for detailed assessment of the features of delirium including severity, duration, and aetiology. It is likely that such variations influence the risk of long-term cognitive impairment [21].

Given the above, there is a need for population-based studies to avoid the selection biases associated with much of the current literature based solely on hospitalised samples [19, 21]. A study that prospectively tracks cognitive change before, during and after delirium would address many of the clinically important questions, including:

- To what extent does delirium influence cognitive outcomes, over and above acute illness and progressing frailty?
- What are the clinical features of delirium that have the greatest impact on cognitive outcomes?
- Are there critical periods where delirium is more harmful?

In a population based cohort study of men and women aged 65 years and older, this study will measure the effect on cognition of an episode of delirium, independent of baseline cognitive status and illness severity. The study will also explore the predictive value of clinical parameters including delirium severity, baseline cognition, and delirium subtype on cognitive outcomes following an episode of delirium.

If this study shows that the relationship between delirium and dementia is highly likely to be independently contributory, it is reasonable to hypothesise that delirium is a potentially modifiable risk factor for dementia. This paves the way for future dementia prevention trials that focus on delirium intervention.

## Methods

### Design

The Delirium and Cognitive Impact in Dementia (DECIDE) study is a nested prospective longitudinal cohort study. The study design, protocol and paperwork have been reviewed and given favourable opinion by the Newcastle and North Tyneside 2 Regional Ethics Committee (REC reference: 15/NE/0353).

### Population

The study is embedded within the Cognitive Function and Ageing Study II-Newcastle centre (CFAS II-Newcastle). This is a large population based cohort of individuals aged ≥65 years at baseline.

At baseline (2008–2011), 2500 participants were recruited using primary care registration, which included care settings, to CFAS II-Newcastle. Participants were re-seen two years later. Global as well as domain specific cognitive function was assessed at baseline and two years follow-up using the Geriatric Mental State (GMS), the Cambridge Cognitive Examination (CAMCOG) and the Mini Mental State Examination (MMSE). All participants sampled in CFAS II-Newcastle live within the catchment area of the Newcastle-upon-Tyne Hospitals NHS Foundation Trust. All surviving CFAS II-Newcastle participants will be eligible to participate.

### Recruitment

At the start of the DECIDE study, an introductory letter and participant information sheet will be sent to all surviving members of CFAS II-Newcastle by the CFAS team detailing the proposed study. They will be invited to contact the CFAS team if they do not want their NHS number to be shared with the DECIDE study team.

During a one-year period, members of CFAS II-Newcastle will be approached on emergency or elective admission to hospital. In order to identify participants admitted to hospital, an alert will be set up on the Newcastle upon Tyne hospitals electronic records system. This will flag up participants on admission to the two acute hospitals in Newcastle upon Tyne (Royal Victoria Infirmary and Freeman Hospital). They will be approached as soon as possible following admission.

### Inclusion criteria

Any participant in CFAS II-Newcastle admitted to hospital during the recruitment period will be invited to take part. If the participant themselves lacks capacity, according to a capacity assessment performed by the lead researcher (SR), an appropriate personal consultee must be available and provide written confirmation of willingness to participate.

### Exclusion criteria

Patients will be excluded from the study if they lack capacity to consent and the study team are unable to identify or contact an appropriate personal consultee. Participants will also be excluded if they are receiving end of life care. If the patient is being isolated for infection control reasons, invitation to participate will be delayed until they are no longer being isolated. Participants in hospital for less than 24 hours will not be included.

### Data collection

The primary exposure is delirium during hospital admission, ascertained prospectively using a standardised procedure based on DSM-5 criteria [22] (Table 1). This assessment combines objective testing of the participant, lasting approximately ten minutes, with information gained from informants (usually nurses, next of kin and clinical records) and assessor’s judgement regarding subjective features. Along with determining whether delirium is present according to DSM-5, the assessment enables scores to be generated for delirium severity, based upon the Memorial Delirium Assessment Scale [23], and motor subtype, based upon the Delirium Motor Subtype Scale [24].

**Table 1:** Standardised diagnostic algorithm for DSM-5 delirium

DSM-5 criteria	Test to be performed or information needed
A. Disturbance in attention (i.e., reduced ability to direct, focus, sustain, and shift attention) and awareness (reduced orientation to the environment).	Observations by the examiner during the interview (initiated by questioning such as “can you tell me what has been going on today?”)
	Level of arousal measured using m-RASS and OSLA
	Months of the year backwards
	Digit span from MDAS
B. The disturbance develops over a short period of time (usually hours to a few days), represents a change from baseline attention and awareness, and tends to fluctuate in severity during the course of a day.	Acute onset and/or fluctuation obtained from informant history from nursing staff, next of kin and clinical notes
C. An additional disturbance in cognition (e.g., memory deficit, disorientation, language, visuospatial ability, or perception).	Impairment in any of the following domains: SHORT-TERM MEMORY: three item recall at three minutes LONG-TERM MEMORY: when did World War II end? ORIENTATION: 10 orientation questions from MDAS LANGUAGE: 3 stage command, naming an object and explain purpose of object along with fluency, comprehension and content of conversation VISUOSPATIAL: Will a stone float on water? PERCEPTUAL DISTURBANCE: evidence of illusions or hallucinations by collateral or direct observation/questioning
D. The disturbances in criteria A and C are not explained by another pre-existing, established, or evolving neurocognitive disorder and do not occur in the context of a severely reduced level of arousal, such as coma.	Information from history/chart/clinical examination
E. There is evidence from the history, physical examination, or laboratory findings that the disturbance is a direct physiologic consequence of another medical condition, substance intoxication or withdrawal (i.e., because of a drug of abuse or to a medication), or exposure to a toxin or is because of multiple aetiologies.	Information from history/chart/clinical examination

Abbreviations:

*m-RASS* modified Richmond Agitation and Sedation Scale [37]

*OSLA* Observational Scale of Level of Arousal [38]

*MDAS* Memorial Delirium Assessment Scale [23]

Participants will be assessed daily for the first five days by SR or a clinical research nurse, both trained in the assessment of delirium. From day 6, those with delirium will continue to be seen daily until delirium resolution. In the absence of delirium from day 6, or following resolution of delirium, participants will be screened regularly for delirium using a semi-structured interview including a modified version of the Delirium Observation and Screening Scale [25]. Should participants display any signs of delirium according to this, the full assessment described above will be performed to determine whether DSM-5 delirium is present.

These assessments will enable the recording of the duration and characteristics of delirium or the development of new delirium. It will be possible to follow the natural history of the delirium in terms of any fluctuations, potential resolution and therefore estimate duration. The subsequent development of delirium in previously non-delirious participants will also be captured. If it is not possible to review participants prospectively at any particular time point, due to illness, refusal or study capacity, a validated tool will be used to retrospectively review the medical records for a diagnosis of delirium [26].

Recruitment of hospital attendees will stop after 12 months. At this point, vignettes will be generated for each participant and these will be sent to an expert consensus panel (LA, SP, and DD). The panel will be tasked with determining whether delirium was present, its severity, duration and subtype. The use of a consensus panel enables an objective approach to determining these factors. Participants will be identified only by their unique study identifier and will therefore be anonymised.

Participants will be invited to the study on every admission during the one year study period.

Illness severity will also be recorded during admission using recognised illness severity measures (APACHE II [27]/SOFA [28]/SAPS II [29]). There are a lack of validated tools to measure illness severity in older people. Therefore, the utility of the HABAM [30] as a surrogate marker for illness severity and recovery along with delirium development/resolution will also be explored.

At random time-points throughout the study, joint assessments of a sample of participants will be completed to monitor inter-rater reliability and to optimise consistency between assessors.

The advantage of using CFAS participants is that the study population are well characterised at baseline. However, participants were last seen several years ago. Therefore, to obtain an up to date estimate of baseline functioning prior to admission, data will be collected on each admission regarding independence (based on Barthel Index of Activities of Daily Living), frailty (Rockwood Clinical Frailty Score [31]), co-morbidities (CIRS-G [32]), nutritional status (Malnutrition Universal Screening Tool and BMI), polypharmacy (number of medications) and anti-cholinergic burden (based on the ACB scale [33]). The predictive value of these parameters will be explored. During admission, possible causes of delirium will also be recorded along with relevant delirium risk factors such as ward moves, dehydration, constipation, pain and presence or absence of sensory aids.

### Outcomes

The primary outcome will be cognitive decline and/or dementia 12 months after hospital discharge, in comparison to pre-delirium cognitive function, measured by the CAMCOG. All participants, with and without delirium, recruited in hospital will be invited for follow up 12 months after their most recent hospital discharge. Follow up will consist of a home visit to complete the Geriatric Mental State-Automated Geriatric Examination for Computer Assisted Taxonomy (GMS-AGECAT) which provides a quantitative measure of cognition along with dementia status and was used in CFAS II. Other relevant data will also be recorded such as place of residence and an assessment of physical function.

A control group of non-hospital attendees without evidence of delirium will be sampled on a 2:1 basis, matched for age, sex and education. Absence of a history of delirium will be ascertained via clinical notes review and telephone interview based on the Informant Assessment of Geriatric Delirium Scale (I-AGeD) [34].

### Consent

Potential participants will be approached as soon as possible on admission to hospital. This will not interfere with clinical care. They will be approached by the chief investigator (SR) or the research nurse. Participants will be given a maximum of 24 hours to decide. However, given the low risk and largely observational nature of the study, it is anticipated that participants or their personal consultees will be willing to consent before this. They will also previously have received a participant information sheet by post and so some participants may already be aware of the study.

The inclusion of some participants lacking capacity is inevitable as the study aims to look at the effect of delirium on cognition and both delirium and dementia can impair a person’s capacity. A formal capacity assessment based on the Mental Capacity Act will be performed by a trained member of the research team, mainly the chief investigator (SR). Participants will be asked to give consent appropriate to their level of understanding, ranging from written informed consent to account being taken of verbal and non-verbal communication in determining willingness to participate. In those individuals found to be without capacity to give full written informed consent, a personal consultee will be identified and their advice sought regarding participation as per Section 32 of the Mental Capacity Act [35]. As per this guidance, the personal consultee cannot be a paid carer.

The advantage of re-evaluating CFAS II participants, as opposed to other study populations, is that they have already expressed an interest in research by virtue of their willingness to participate in CFAS II. This may make conversations with the personal consultee easier as they will be familiar with research and should be aware of the wishes and feelings of the participant about taking part in research studies.

Verbal reconfirmation of the study participant’s willingness to continue with the study will be sought at each point of contact. Participants who are very distressed or refuse to engage (whether due to delirium or having the capacity to refuse assessment on that occasion) will not be assessed by the research team on that occasion but a record of the outcome of the interaction will be documented. Due to the fluctuating nature of delirium, further contact will be attempted later. Any patient appearing consistently distressed by participation or withdrawing consent whilst having capacity will be excluded from the study without prejudice to clinical care. As such, every effort will be made to respect the wishes of the person, both previously made and at the time the research is undertaken.

If they recover capacity, participants admitted to the study via a personal consultee will be given the opportunity to consider whether they would like to continue to be part of the study and if so, written consent will be obtained.

### Data handling and confidentiality

Data will be handled, computerised and stored in accordance with the Data Protection Act 1998. No participant identifiable data will leave the study site. Participants will be allocated a unique study identifier which will be used on all data stored in order to ensure anonymity. Caldicott approval has been granted. Personal data will be regarded as strictly confidential. All study records, including the consent forms, and investigator site files will be kept in a locked filing cabinet with restricted access.

### Power calculations

We aim to detect a clinically and statistically significant difference in the annual decrease in the total CAMCOG (total score 107), between 6 points in delirium participants and 3 points in participants without delirium. Assuming that 10% of the cohort (the most conservative end of the range) is a delirium case and that the standard deviation of the decrease in CAMCOG is 2.7 points [36], then in order to detect the desired difference (2-fold) in the CAMCOG with 90% power, using a two-sided test at the 5% level, 10 participants with delirium at the time of admission to hospital and 90 participants without delirium would be needed, i.e. a total of 100 participants. The analysis would additionally allow for other factors using a regression approach, rather than simply comparing how the change in CAMCOG from admission to follow-up differs between the two groups. Also, whilst the calculation assumes complete data have been collected for all participants, the analysis will explore the possibility of incorporating participants with missing data. The above calculations are based on a very conservative prevalence of delirium of 10%. If, for example, the prevalence were 20%, then a total of 55 participants (11 with delirium, 44 without) would be required, based on the assumptions outlined above.

By applying the expected number of admissions per age group, based on best available data, to the number of people within these age groups remaining in the CFAS II-Newcastle cohort, it is possible to estimate that 450 people will be admitted during the year.

### Statistical analysis

The CAMCOG will be used as the primary measure of global cognitive status when examining the effect on cognition of an episode of delirium. The distribution of the values will be considered during the analysis process. The effect of an episode of delirium on cognition will be evaluated by comparing CAMCOG score at baseline and at one year after admission to hospital. Regression analyses will be used to evaluate the change in cognition in delirious and non-delirious participants whilst accounting for relevant confounders such as age, education and illness severity.

Drop-out due to mortality is anticipated in both groups due to the age of participants and the high mortality rates associated with both severe illness and delirium. This will be explored as part of the analysis. The sensitivity of our results to patterns of missing data and methods for accounting for this will be explored. There is likely to be some survivor effect and this will be considered. Longitudinally, the labelling of “delirium” and “control” becomes blurred due to the fluctuating nature of delirium. The possibility of analysing delirium as a time-varying exposure will be explored as part of the data analysis. The overall approach is novel because no previous delirium ascertainment studies have been nested within an existing, well-characterised cohort allowing baseline characteristics to be controlled for in the final analysis. Assistance with data analysis will be sought from collaborators who have experience in this field and have also previously worked with the CFAS-II cohort.

The predictive value, in terms of cognitive outcomes, of the various markers recorded during the acute episode will be evaluated using multiple regression analysis. This might include the contributory and/or independent effects of delirium severity, duration, aetiology or baseline cognitive function on cognitive outcome.

## Discussion

Delirium is common and associated with poor outcomes, but existing studies are limited by a lack of generalisability and the use of retrospective delirium ascertainment. The novel design of this population-based study includes both baseline cognitive assessments and prospective delirium evaluation in order to assess robustly the likelihood of the relationship between delirium and cognitive decline being independent of any potentially confounding factors. This study will elucidate the size of the effect of delirium upon cognitive decline/dementia and may lead on to dementia prevention trials that focus on delirium intervention. Given the current lack of both modifiable risk factors and treatments for dementia, this would be a considerable advance. Validated methods of delirium prevention do exist but have not been widely implemented. This study will add to our understanding of the long-term importance of delirium prevention.

The prospective nature of the delirium assessments, and the data to be collected, will increase our understanding of the natural history of delirium. As such, this study could address many unanswered questions of clinical significance in delirium and dementia including the natural history of delirium, expected outcomes, and the prognostic value of parameters such as aetiology, duration, severity or underlying frailty. This would facilitate accurate and realistic conversations with families and will have important implications for healthcare planning and public health initiatives.

Patients and the public have been involved throughout the development of this study. Patient groups via the Dementias and Neurodegenerative Diseases Research Network (DeNDRoN) were consulted regarding study design, particularly in terms of acceptability. They also reviewed the lay summary. The proposal was also reviewed by lay members of the Cognitive Function and Ageing Study (CFAS) Management Committee. They supported the application and agreed that the combination of the two studies will be reciprocally beneficial with DECIDE enriching CFAS data and vice versa. As the study progresses, regular meetings with the Alzheimer’s Society monitors will be used to provide updates on progress and to disseminate findings. There will also be an open Patient and Public Involvement dissemination event at the end of the study.

DECIDE will be the first study to prospectively elucidate the effect of delirium upon cognitive decline and dementia independent of baseline cognition and illness severity and may inform future dementia prevention trials that focus on delirium intervention.

## Abbreviations

CFAS II-Newcastle: Cognitive Function and Ageing Study II Newcastle cohort
DECIDE: Delirium and Cognitive Impact in Dementia
GMS-AGECAT: Geriatric Mental State-Automated Geriatric Examination for Computer Assisted Taxonomy
CAMCOG: Cambridge Cognitive Examination
MMSE: Mini Mental State Examination
DSM-5: Diagnostic and Statistical Manual of Mental Disorders 5th edition [22]
APACHE II: Acute Physiology and Chronic Health Evaluation II [27]
SOFA: Sepsis-related Organ Failure Assessment [28]
SAPS II: Simplified Acute Physiology Score [29]
HABAM: Hierarchical Assessment of Balance and Mobility [30]
CIRS-G: Illness Rating Scale – Geriatrics [32]
BMI: Body Mass Index
ACB: anti-cholinergic burden
I-AGeD: Informant Assessment of Geriatric Delirium Scale [34]
DeNDRoN: Dementias and Neurodegenerative Diseases Research Network
m-RASS: modified Richmond Agitation and Sedation Scale [37]
OSLA: Observational Scale of Level of Arousal [38]
MDAS: Memorial Delirium Assessment Scale [23]
